# A Role of Fluoride on Free Radical Generation and Oxidative Stress in BV-2 Microglia Cells

**DOI:** 10.1155/2012/102954

**Published:** 2012-08-13

**Authors:** Xi Shuhua, Liu Ziyou, Yan Ling, Wang Fei, Guifan Sun

**Affiliations:** Department of Environmental and Occupational Health, Liaoning Provincial Key Laboratory of Arsenic Biological Effect and Poisoning, School of Public Health, China Medical University, District of Heping, North Er Road No. 92, Shenyang 110001, China

## Abstract

The generation of ROS and lipid peroxidation has been considered to play an important role in the pathogenesis of chronic fluoride toxicity. In the present study, we observed that fluoride activated BV-2 microglia cell line by observing OX-42 expression in immunocytochemistry. Intracellular superoxide dismutase (SOD), glutathione (GSH), malondialdehyde (MDA), reactive oxygen species (ROS), superoxide anions (O_2_^∙−^), nitric oxide synthase (NOS), nitrotyrosine (NT) and nitric oxide (NO), NOS in cell medium were determined for oxidative stress assessment. Our study found that NaF of concentration from 5 to 20 mg/L can stimuli BV-2 cells to change into activated microglia displaying upregulated OX-42 expression. SOD activities significantly decreased in fluoride-treated BV-2 cells as compared with control, and MDA concentrations and contents of ROS and O_2_^∙−^ increased in NaF-treated cells. Activities of NOS in cells and medium significantly increased with fluoride concentrations in a dose-dependent manner. NT concentrations also increased significantly in 10 and 50 mg/L NaF-treated cells compared with the control cells. Our present study demonstrated that toxic effects of fluoride on the central nervous system possibly partly ascribed to activiting of microglia, which enhanced oxidative stress induced by ROS and reactive nitrogen species.

## 1. Introduction

Fluoride is an ubiquitous element in the environment and has a remarkable prophylactic effect at low concentrations by inhibiting dental caries, while at higher concentrations it causes dental and skeletal fluorosis [1]. Endemic fluorosis is prevalent in many parts of the world and causes damage not only to hard tissues of teeth and skeleton, but also to soft tissues, such as brain, liver, kidney, and spinal cord [2]. Epidemiological investigations reveal that intelligence quotient (IQ) of children living in endemic fluorosis areas is lower than that of children living in low fluoride areas [3–7]. It has been demonstrated that high concentrations of fluoride can decrease learning ability and memory in some animal experiments [8, 9] and result in dysfunctions of the central nervous system (CNS) [10, 11]. As the cases of many chronic degenerative diseases, the increase of reactive oxygen species (ROS) and lipid peroxidation (LPO) has been considered to play an important role in the pathogenesis of chronic fluoride toxicity [12–14]. Fluoride administration significantly increases brain LPO level compared with control group in rat, while reduced glutathione (GSH) content and superoxide dismutase (SOD), glutathione peroxidase (GPx), and glutathione reductase (GR) activities decrease markedly in fluoride-treated groups [15, 16]. There are significantly negative correlations between fluoride concentrations in brain and GPx activity, GSH level, and positive correlations between fluoride concentrations and thiobarbituric acid reactive substances (TBARSs) and carbonyl groups [17].

The CNS is especially sensitive to free radical oxidative damage as it contains more easily oxidizable fatty acids [18, 19]. ROS is produced during the respiratory burst of phagocytes, and the regulated generation of ROS plays an important role in host defense, oxygen sensing, and signal transduction [20, 21], while excessive production ROS promotes cellular injure and tissue damage. Macrophages are sources of free radicals, including ROS and reactive nitrogen species (RNS). Microglia are a kind of resident macrophage of the CNS and play a vital role in immune surveillance and injury repair [22, 23]. Microglia activation is a common phenomenon in response to exposure to toxicants, and activated microglia are both phagocytic and potent sources of reactive oxygen and nitrogen intermediates [24–26]. Microglia excessive activation also can trigger or exacerbate neurotoxicity by inducing oxidative stress of neurons [27]. Nitric oxide (NO) production results from nitric oxide synthase (NOS) that catalyze the conversion of L-arginine to L-citrulline and NO. At high concentrations, NO readily reacts with superoxide anion (O_2_^∙−^), a kind of ROS derived from nicotinamide adenine dinucleotide phosphate (NADPH) oxidase (NOX), to produce peroxynitrite (ONOO^*‒*^). ONOO^*‒*^ is able to irreversibly inhibit mitochondrial respiration, react with proteins, lipids, carbohydrates and DNA, and cause DNA fragmentation and lipid oxidation. 

A growing number of studies have shown that fluoride can increase the generation of ROS and LPO in brain [15], but it is not known if ROS increasing in brain is related with activated microglia at fluoride exposure. In the present study, we treated BV-2 microglia cell line with different concentrations of fluoride and found that BV-2 microglia cells were activated. The levels of ROS and RNS were increased. The results indicated that activating BV-2 microglia cells by fluoride induced oxidative stress, which provides a potential oxidative stress mechanism for fluoride-related brain damage. 

## 2. Materials and Methods 

### 2.1. Chemicals and Reagents

Sodium fluoride (NaF, molecular weight 41.99) was procured from Sigma Chemical (St. Louis, MO, USA). All other analytical laboratory chemicals and reagents were obtained from Sigma, Invitrogen (Carlsbad, CA, USA), Hyclone (Logan, UT, USA). 

### 2.2. Cell Culture and Treatment

The immortalized murine microglia cell line, BV-2, was provided by Cell Culture Center, School of Basic Medicine, Peking Union Medical College. The BV-2 cells were maintained in Dulbecco's modified Eagles medium (DMEM) that contained 10% fetal bovine serum and antibiotics at 37°C in a 5% CO_2_ humified incubator. Exponentially growing BV-2 cells at density of 5000 cells in 100 *μ*L medium were treated with 0.5–120 mg/L NaF respectively for 24, 48, and 72 h for cell viability assay. The concentrations of NaF (1, 10, and 50 mg/L) were selected for intracellular SOD, GSH, malondialdehyde (MDA), ROS, O_2_^∙−^, NOS, Nitrotyrosine (NT) measurement and cell medium was collected for NO, NOS assay.

### 2.3. MTT Cell Viability Assay

Viability and growth patterns of BV-2 cells based on mitochondrial enzyme functions in 96-well plates were determined under NaF-treated for 24 h, 48 h, and 72 h by 3-[4,5-dimethylthiazol-2-yl]-2,5-diphenyltetrazolium bromide (MTT) conversion to formazan. Briefly, 100 *μ*L of MTT solution (0.5 mg/mL in the medium) was added to each well, and the plates were incubated at 37°C for additional 4 h. Afterwards, the medium containing MTT was removed, and the crystals were dissolved in 150 *μ*L of 100% dimethyl sulfoxide (DMSO). The cell viability was quantified using a microplate reader (Multiscan Ascent, Labsystems, Finland) at 570 nm.

### 2.4. Immunocytochemistry

Following NaF treatment, cultured microglia were fixed with 4% paraformaldehyde and incubated with the diluted primary antibody (1 : 200 OX-42, microglia marker) for 20 min at 37°C, and secondary antibody sheep anti-mouse IgG was added for 20 min at 37°C. After washing, the cells were incubated with DAB dilution until an observed color change. 100 ng/mL Lipopolysaccharides (LPS) was as a positive control. Staining of OX-42 was examined at high-power fields (× 400) under a standard light microscope. The number of cells as identified by OX-42 antibody was counted in five different fields on each microscope slide. 

### 2.5. Determination of Intracellular GSH, SOD, and MDA

BV-2 cells were cultured in 10 cm diameter dishes and treated with NaF as indicated. At the end of treatment, cells were washed 3 times with ice-cold phosphate-buffered saline (PBS), scraped off the dishes with a silicone “policeman” and harvested into Eppendorf tubes. Then cells were lyzed in PBS by sonication and centrifuged at 15000 ×g for 5 min at 4°C. The resulting supernatants were used immediately for the measurement. The intracellular GSH levels and SOD activity were measured with improved 5,5′-dithiobis-(2-nitrobenzoic acid) (DTNB) method and hydroxylamine assay, respectively, provided by commercial test kits (KeyGen Biotech. Co. Ltd., Nanjing, China). The LPO was assessed by measuring MDA levels. The quantification was based on measuring formation of thiobarbituric acid reactive substances (TBARS) according to the manufacturer's protocol. Protein concentrations were determined by the method of Bradford [28] to normalize the levels of GSH, SOD, and MDA. The results were expressed as mg/g protein for GSH, U/mg protein for SOD, and nmoL/mL for MDA.

### 2.6. Measurement of Intracellular ROS and O_2_^∙−^  Generation

The production of ROS was determined by measuring dichlorofluorescein (DCF) fluorescence as described by Rothe and Valet [29]. Briefly, BV-2 cells in 24-well culture plates, treated with NaF for 24 h, were rinsed and resuspended in serum-free medium containing 10 *μ*M DCFH-DA. After a further 30 min of incubations at 37°C, cells were rinsed twice with ice-cold PBS and harvested by trypsin, and were measured via a FACScan flow cytometer (Becton Dickinson, USA) with excitation wave length at 488 nm and emission wave length at 525 nm for ROS generation. The fluorescence intensity parallels to the amount of ROS formed. Intracellular O_2_^∙−^ was detected by measuring dihydroethidium (DHE) fluorescence. Briefly, BV-2 cells were harvested after treated with NaF for 24 h, washed with serum-free culture medium and incubated with 5 *μ*M DHE at 37°C for 30 min. Then the cells were harvested, washed and resuspended, and were measured via a FACScan flow cytometer (BECTON DICKINSON, USA) with excitation wave length at 300 nm and emission wave length at 610 nm for O_2_^∙−^ generation. 

### 2.7. Determination of NO Release and NOS

BV-2 cells were treated with various concentrations of NaF for 24 h. The cell culture medium was collected, then centrifuged 1 min at 13000 rpm to remove floating cells and cell debris, and analyzed immediately for nitrite content. The production of NO was determined by measurement the nitrite accumulation in the medium using colorimetric assay with Griess reagent. Nitrite concentrations were determined by comparison with standard solutions of sodium nitrite at optical density 550 nm.

BV-2 cells and medium were collected after treated with NaF as indicated. Then cells were lyzed in PBS by sonication and centrifuged at 15000 ×g for 5 min at 4°C, the resulting supernatants and medium were used as sources of NOS, respectively. NOS activity was determined by monitoring the formation of [3H] L-citrulline from [3h] L-arginine as the method described previously [30]. The commercial test kit of NOS was provided by Nanjing Jiancheng Biological Corporation (Nanjing, China).

### 2.8. Enzyme-Linked Immunosorbent Assays (ELISA)

BV-2 cells were treated with various concentrations of NaF for 24 h, and the intracellular NT was assessed by ELISA using monoclonal antibodies and the procedure recommended by the supplier (Abway Antibody Technology Co., Ltd., Beijing, China). The concentration of NT was calculated according to the standard curve of the ELISA kits.

### 2.9. Statistical Analysis

All experiments were performed at least in triplicate and the values represent mean ± SD. Differences between groups were statistically analyzed by one-way analysis of variance (ANOVA) and positive rates of OX-42 expression were analyzed by the Fisher Exact test. Differences between experimental groups with a *P* value <0.05 were considered significant. 

## 3. Results 

### 3.1. Fluoride Decreased the Cell Viability of BV-2 Cells

**Figure 1:**
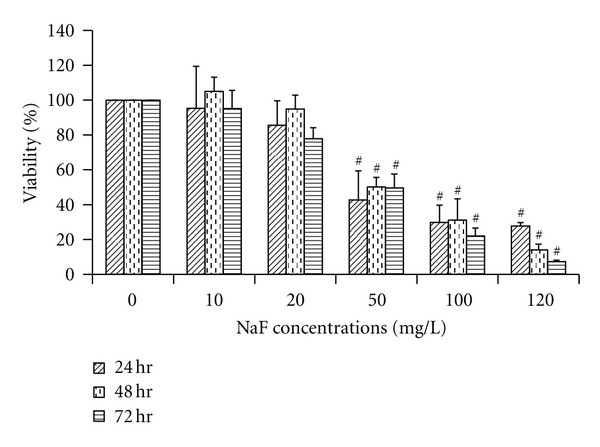
Effects of fluoride on cell viability in microglial BV-2 cells. Cells were treated with various concentrations of NaF and incubated for 24, 48, and 72 h. Cell viabilities were measured using MTT assay, and data were presented as a percentage of the control. ^#^ *P* < 0.01 compared to the control group.

As shown in Figure 1, fluoride reduced the BV-2 cell viability in 50, 100, or 120 mg/L NaF groups. At concentrations of 50, 100, or 120 mg/L, sodium fluoride caused a significant decrease in cell viability of 57%, 70%, and 72%, respectively, versus control at NaF-treated 24 h, 50%, 69%, and 86%, respectively, versus control at NaF-treated 48 h, and 51%, 78%, and 92%, respectively, versus control at NaF-treated 72 h.

### 3.2. BV-2 Cell Activation after Treatment with Fluoride

**Figure 2:**
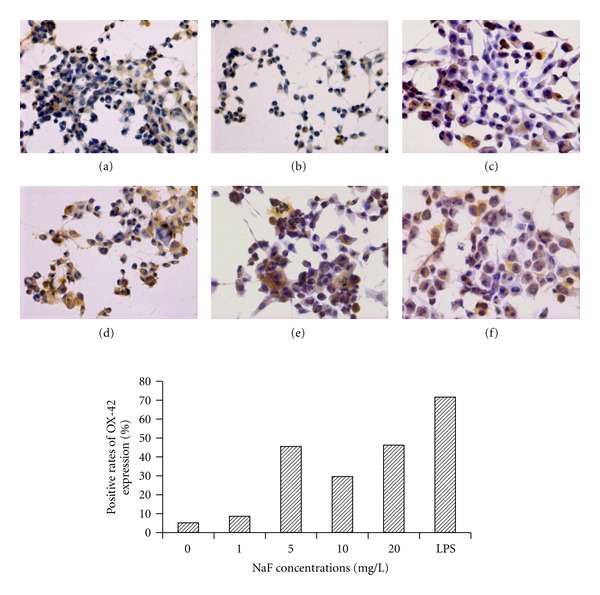
The activity of microglial BV-2 cells induced by fluoride. Cells were treated with indicated concentrations of NaF for 24 h and immunocytochemistry localization with an OX-42 antibody as a microglial marker were observed. Microglial activity was detected by OX-42 expression, and the microglia cells treated with LPS were used as a positive control. Morphological changes of microglia from the resting state ((a) small cell bodies and thin, long, or ramified processes) to the activated state ((c), (d), (e), (f) larger cell bodies with short, thick) were observed after fluoride or LPS treatment in the BV-2 cells. High expression areas of OX-42 immunoreactivity were indicated by arrows. Optic microscopy: HE (400×). (a) control, (b) 1 mg/L NaF, (c) 5 mg/L NaF, (d) 10 mg/L NaF, (e) 20 mg/L NaF, and (f) 100 ng/mL LPS.

Integrin *α*M (OX-42) is a cell adhesion molecule that acts as a receptor for cell surface ligands. Immunocytochemistry localization with an OX-42 antibody was used as a microglial marker. To confirm the activation of BV-2 in response to NaF, OX-42 expression was determined by immunocytochemistry after 24 h incubation with different NaF concentrations (1–20 mg/L). NaF of concentrations from 5 to 20 mg/L can stimulate BV-2 cells to change into activated microglia displaying upregulated OX-42 expression and large bodies compared with control microglia with small bodies and long branch (Figure 2). The number of cells showing OX-42 positive expression markedly increased in 5, 10, and 20 mg/L NaF-treated BV-2 cells compared with the control.

### 3.3. Effects on the Levels of SOD, GSH, MDA, and ROS of Fluoride in BV-2 Cells

**Figure 3:**
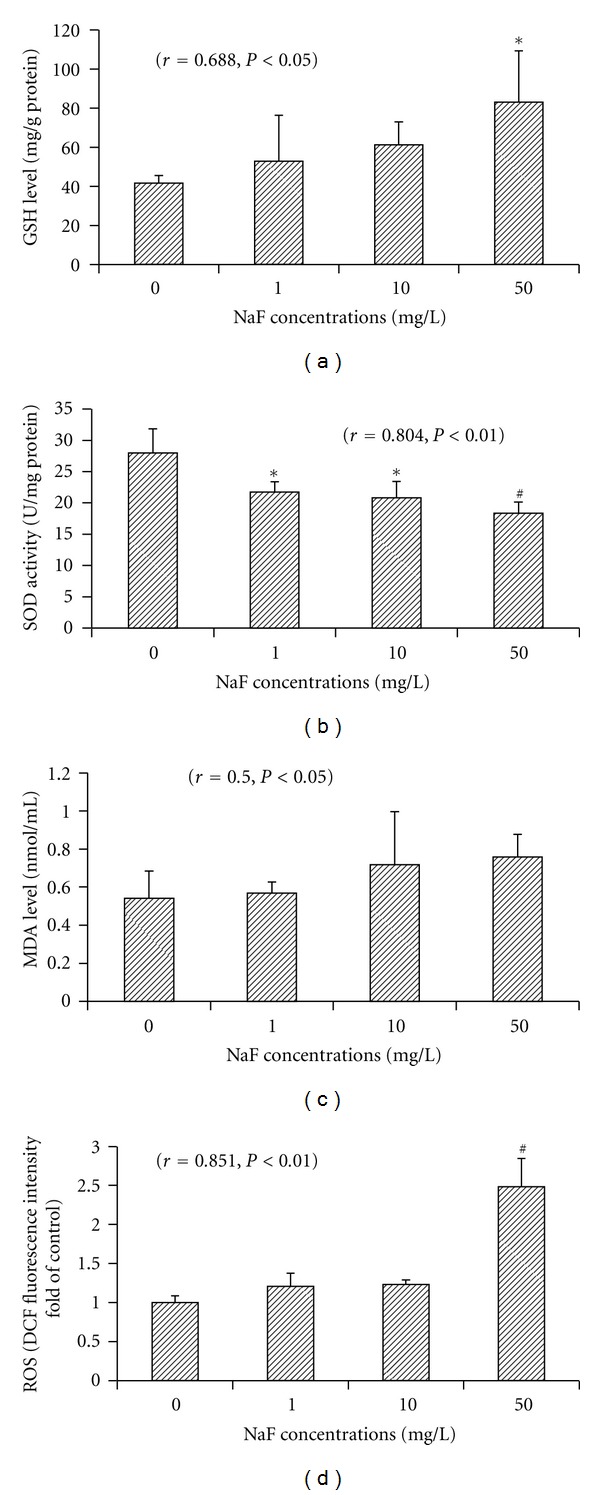
Effects of fluoride on intracellular GSH levels (a); SOD activity (b); LPO production (MDA) (c) and ROS production (d) in microglial BV-2 cells. After the cells were treated with various concentrations of NaF (1, 10, and 50 mg/L) for 24 h, the contents of GSH, MDA and the activities of SOD were measured using commercial test kits. DCF fluorescence intensity was measured by flow cytometry for ROS content and DCF fluorescence intensity fold of control was analyzed. Bars were presented as mean ± SD. *P < 0.05 and ^#^ *P* < 0.01 compared to the control group.

As shown in Figure 3, a marked decrease in SOD activities was observed in fluoride-treated BV-2 cells. SOD activities decreased significantly by 22.3%, 25.6%, and 34.4% in 1, 10, and 50 mg/L NaF-treated cells, respectively, compared to the control cells, and there was a significant negative dose-effect relationship (*r* = 0.804, *P* < 0.01). The content of intracellular ROS was evaluated by the changes in DCF fluorescence intensity. DCF fluorescence intensities in BV-2 cells increased with fluoride concentrations, with a significant dose-dependent manner (*r* = 0.851, *P* < 0.01), and the DCF fluorescence intensities in the 50 mg/L NaF-treated cells were significantly higher than the control cells (*P* < 0.01). MDA concentrations also increased in NaF-treated cells, and there was a significant positive dose-effect relationship (*r* = 0.5, *P* < 0.05) although there were no statistical differences in NaF-treated cells compared with the control cells. However, there was an increase tendency in NaF-treated cells compared to the control for the intracellular GSH levels.

### 3.4. Effects on NOS Activity and NO Concentration of Fluoride in BV-2 Cells

**Figure 4:**
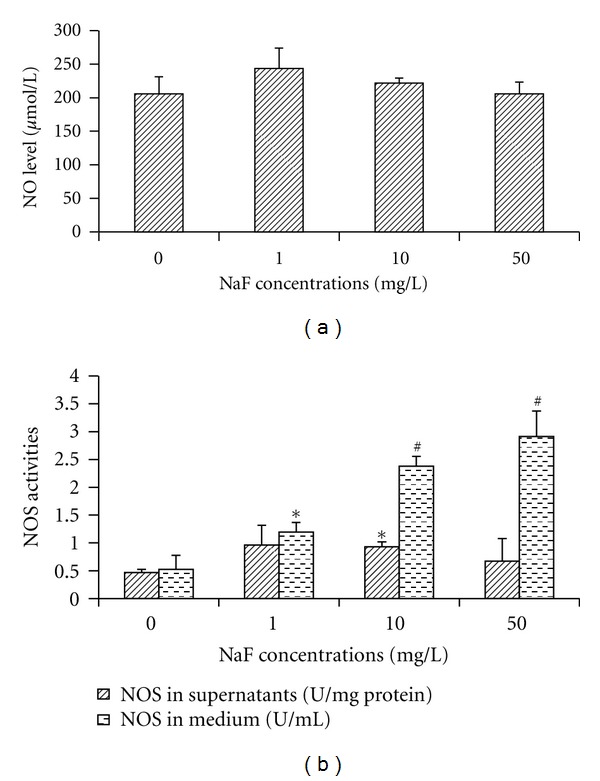
Effects of fluoride on NO production (a), and NOS activities (b) in microglial BV-2 cells. After the cells were treated with various concentrations of NaF (1, 10, and 50 mg/L) for 24 h, NO release in culture medium and NOS activities in supernatants and medium were measured using commercial test kits. Bars were presented as mean ± SD. *P < 0.05 and ^#^ *P* < 0.01 compared to the control group.

In addition to ROS, RNS from activated microglia can be a major cause of oxidative stress. We therefore measured NO and NOS in BV-2 cells treated with various concentrations of fluoride for 24 h. NO level in the culture medium did not increase with fluoride concentrations. However, activities of NOS, synthesizing NO, significantly increased in 1, 10, and 50 mg/L NaF-treated BV-2 cell culture medium compared with the control, in a dose-dependent manner (*r* = 0.96, *P* < 0.01). Intracellular NOS activities only increased in 10 mg/L NaF-treated BV-2 cells compared with the control, see Figure 4.

### 3.5. The O_2_^∙−^  and NT Level in NaF-Treated BV-2 Cells

**Figure 5:**
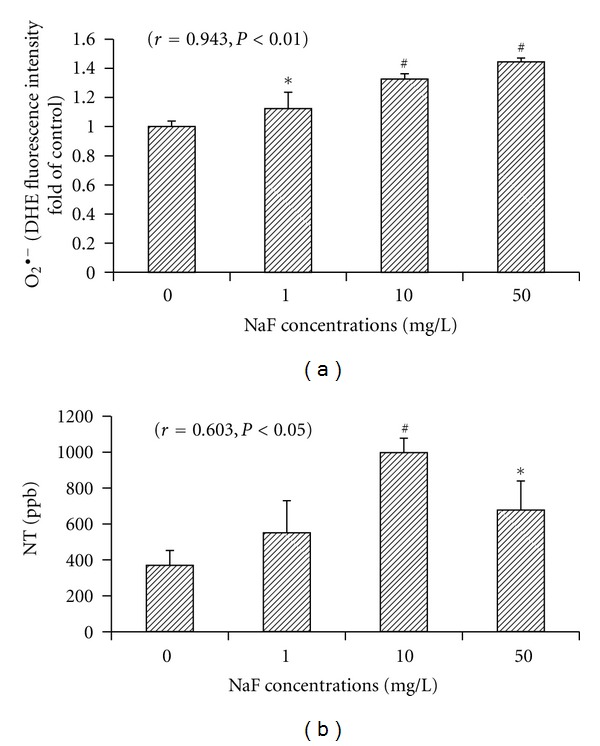
Effects of fluoride on O_2_^∙−^ production (a) and NT concentrations (b) in microglial BV-2 cells. BV-2cells were treated with various concentrations of NaF (1, 10, and 50 mg/L) for a 24 h incubation period, and DHE fluorescence intensity was measured by flow cytometry for O_2_^∙−^ content and DHE fluorescence intensity fold of control was analyzed. Intracellular NT concentrations were measured by ELISA. Bars were presented as mean ± SD. *P < 0.05 and ^#^ *P* < 0.01 compared to the control group.

The content of intracellular O_2_^∙−^ was determined by the changes in DHE fluorescence intensity. DHE fluorescence intensities in BV-2 cells increased with fluoride concentrations, with a significant dose-dependent manner (*r* = 0.943, *P* < 0.01), and the DHE fluorescence intensities in the 1, 10, and 50 mg/L NaF-treated cells were significantly higher than the control cells. Peroxynitrite, the reaction product of NO and O_2_^∙−^, can lead to macromolecule damage and formation of NT, a protein adduct, and NT has been as a marker of free radical oxidation of nitric oxide. Our study showed that intracellular NT concentrations in BV-2 cells increased significantly following fluoride exposure in 10 and 50 mg/L NaF-treated groups compared with the control group, and there also existed a dose-dependent enhancement (*r* = 0.603, *P* < 0.05), see Figure 5.

## 4. Discussion 

The redox balance of the cell maintained by oxidants and antioxidants is important to physiological activity and function. Disturbance of the natural equilibrium of free radical over generation causes dysfunction and promotes oxidative damage to tissue. Although ROS have some essential roles in normal cell functions [31], they are more associated with their pathological effects that ultimately lead to protein and cellular damage as well as cell death [32, 33]. ROS could attack numerous cell components like membrane lipids, resulting in enhanced lipid peroxidation and cellular toxicity. ROS has been related with the pathologies of over one hundred diseases. Many studies [34, 35] have reported that excessive fluoride exposure can damage the redox balance of the cells in tissues, decrease antioxidant defense capacity in brain [15], and increase the toxic effects on visceral organs mediated by generation of ROS and lipid peroxidation [36]. A close association between chronic fluoride toxicity and increased oxidative stress has been reported in humans and in experimental animals [37, 38]. Neurotoxicity induced by fluoride had been linked with oxidative stress [11, 16].

Microglia are the resident immune cells in the brain and readily activated by a variety of foreign substances including environmental toxicants [39]. The beneficial roles of microglia activation include clearance of toxic cell debris and pathogens, induction of innate immune responses, and enhancement of neuronal survival. However, an array of ROS and RNS produced and secreted from activated microglia has been believed to be involved with neurotoxicity in brain disorders [40–42]. In the present study, we also observed that ROS increased in fluoride-treated microglia BV-2 cells. Several in vivo studies have reported microglial proliferation and activation early after various brain insults [43]. Thus, it can be hypothesized that oxidative stress of microglia could be one of the causative factors for fluoride induced brain toxicity. Once activated, microglia undergo morphological changes as well as phenotypic alterations in some molecules. Microglial activation is generally measured by a gradual change in morphology from a quiescent ramified form (resting state) to an amoeboid form (activated state) [44], and upregulation of cell surface receptors, such as CD11b (OX-42) [45]. In this study, the majority of NaF-treated BV-2 microglial cells displayed an activated morphology and increased the expression of OX-42 receptor, an abundance surface receptor in activated microglia. The increased number of OX-42 microglia observed in this study may be due to the reactive response of microglia to fluoride treatment. To our knowledge, this is the first time to verify that microglia are activated by fluoride.

The characteristic features of microglial activation are NOX activation and oxidative burst activity increasing. NOX is a superoxide producing enzyme system, the major source of ROS in microglia [46–48]. Superoxide anions (O_2_^∙−^) derivated from NOX can be catalyzed into the hydrogen peroxide (H_2_O_2_) and molecular oxygen by SOD. SOD accelerates O_2_^∙−^ to H_2_O_2_, which can be said as primary defence, as it prevents further generation of free radicals. In the present study, the SOD activity significantly decreased in dose-dependent manner and O_2_^∙−^ content increased in fluoride-treated BV-2 microglia. It can be explained that O_2_^∙−^ generation consumed SOD. Superoxide radicals especially cause oxidation of –SH groups in protein, and it has been demonstrated that thiolate radicals are removed by GSH [49]. GSH also participates in the reaction that destroys hydrogen peroxide, or organic peroxide, free radicals, and certain foreign compounds. GSH is as a direct free radical scavenger, as a cosubstrate for GPx activity and as a cofactor for many enzymes and forms conjugates in endo- and xeno-biotic reactions [50]. The decrease in GSH level is often observed due to increased utilization in oxidative stress. In contrast, GSH levels in BV-2 microglial cells increased with fluoride concentrations in our present study. Oxidative stress is defined as a disturbance in the balance between the production of free radicals and antioxidant defense, and the later is more compensatory [14]. Our observed increase of GSH may be a compensation increase to scavenge excessive superoxides and protect cells against oxidative stress. This may be part of the general physiological response of the body to ROS.

The production of lipid peroxidation, such as MDA, was accumulated as a result of membrane disruption, elicited by the oxidation of poly unsaturated fatty acids of the bilayer. The present study found that the MDA levels showed an increasing tendency in NaF-treated BV-2 cells although there were no significant differences between NaF-treated BV-2 cells and control cells. However, ROS level in 50 mg/L NaF-treated group increased significantly, which indicated that ROS could attack membrane lipids and result in lipid peroxidation. 

NO is a compound of special interest released by activated microglia and has been described in vitro situations to be an important mechanism by which microglia cause neuronal death [51–53]. NO is produced from L-arginine and molecular oxygen by the action of NOS [54, 55] in activated microglia and contribute to the oxidative stress accompanying the inflammatory process [56]. NOS is the key enzyme for NO production and is quantitatively induced in activated glial cells after exposure to stimulators. The neurotoxic effects of NO are generally attributed to its reaction with O_2_^∙−^ to form ONOO^−^, an extremely active oxidizing agent, which can lead to macromolecule damage and form the protein adduct, such as NT, and NT has been as a marker of free radical oxidation of nitric oxide. In the present study, fluoride significantly increased the NOS activity in activated BV-2 cells, and which should produce more NO. Interestingly, in our experiment, NO level did not increase after BV-2 cells were exposed to fluoride, that could due to produced NO reacted rapidly with O_2_^∙−^ to form ONOO^−^. Indeed, we have observed a significant increase of NT level at 10 and 50 mg/L NaF-treated cells, suggesting that neurotoxicity of fluoride is associated with overproduction of nitric oxide and peroxynitrite. Therefore, RNS, together with ROS produced by activated microglia was involved in oxidative stress induced by fluoride in activated BV-2 cells. 

In conclusion, a main finding of this study was that microglia BV-2 cells were activated by fluoride, and the increase of ROS and RNS in microglia was associated with the activation of microglia. The novelty of this work is related to the active role of microglia treated with fluoride. We provided new evidence from in vitro model indicating that fluoride exerts its toxic effects in CNS possibly partly ascribed to activating of microglia in vitro, which enhanced oxidative stress induced by ROS and RNS. Of course, the detailed effect and mechanism need further investigation in vivo.
